# MULTIDIMENSIONALLY HETEROGENEOUS HEALTH LATENT CLASSES AND HEALTHCARE UTILIZATION FOR OLDER CHINESE

**DOI:** 10.1093/geroni/igz038.2542

**Published:** 2019-11-08

**Authors:** Linglong Ye, Jiecheng Luo, Ben-Chang Shia, Ya Fang

**Affiliations:** 1 School of Public Health, Xiamen University, Xiamen, China; 2 School of Management, Taipei Medical University, Taipei, Taiwan

## Abstract

Objectives: Based on a multidimensional perspective, this study aimed to assess the heterogeneous health latent classes of older Chinese, and further examined the effects of health latent classes and associated factors on healthcare utilization. Methods: Data came from the Chinese Longitudinal Healthy Longevity Survey in 2014. Latent class analysis was adopted to identify heterogeneous health latent classes by health indicators of physical, psychological, and social dimensions. Two-part models were used to evaluate the impact of health latent classes and socio-demographic factors on outpatient and inpatient utilization. Results: Among 2,981 participants aged 65 and over without missing health indictors, four health latent classes were identified and labeled as “Lacking Socialization” (10.4%), “High Comorbidity” (16.7%), “Frail Group” (7.7%), and “Relatively Healthy” (65.1%). Among 1,974 participants with complete information, compared with the Relatively Healthy group, those in the Lacking Socialization group costed more inpatient expenditure (p-value =0.02). Those in the High Comorbidity and Frail groups tended to use healthcare services and costed more outpatient expenditure (all p-value <0.01). After controlling for health latent classes, the effects of age, gender, marital status, education, residence area, occupation, and health insurance on healthcare utilization were significant. Conclusions: Four heterogeneous health latent classes were identified by multidimensional health, and had significant effects on healthcare utilization. After controlling for health latent classes, different effects of socio-demographic factors on healthcare utilization were found. It enhances our understanding of heterogeneous health and complex healthcare demands in older Chinese, and is valuable for improving healthcare resource allocation targeted for healthy aging.

